# *Nigella sativa*-Manganese Ferrite-Reduced Graphene Oxide-Based Nanomaterial: A Novel Adsorbent for Water Treatment

**DOI:** 10.3390/molecules28135007

**Published:** 2023-06-26

**Authors:** Nusrat Tara, May Abdullah Abomuti, F. M. Alshareef, Omeima Abdullah, Esam S. Allehyani, Saif Ali Chaudhry, Seungdae Oh

**Affiliations:** 1 Environmental Chemistry Research Laboratory, Department of Chemistry, Jamia Millia Islamia, New Delhi 110025, India; taranusrat.nt@gmail.com; 2 Department of Chemistry, Faculty of Science and Humanities, Shaqra University, Dawadmi 17472, Saudi Arabia; maabumoati@su.edu.sa; 3 Chemistry Department, Faculty of Sciences, King Abdulaziz University, Jeddah 21589, Saudi Arabia; fmalshareef@kau.edu.sa; 4 Pharmaceutical Chemistry Department, College of Pharmacy, Umm Al-Qura University, Makkah 21955, Saudi Arabia; oaabdullah@uqu.edu.sa; 5 Department of Chemistry, University College in Al-Jamoum, Umm Al-Qura University, Makkah 21955, Saudi Arabia; esklehyani@uqu.edu.sa; 6 Department of Civil Engineering, College of Engineering, Kyung Hee University, Gyeonggi-do, Yongin-si 17104, Republic of Korea

**Keywords:** methylene blue, water pollution, wastewater treatment, adsorption, integrated surface, biocompatible material

## Abstract

In this study, a novel nanohybrid composite was fabricated via the incorporation of manganese ferrite (MnFe_2_O_4_) nanoparticles into the integrated surface of reduced graphene oxide (rGO) and black cumin seeds (BC). The nanohybrid composite was prepared by a simple co-precipitation method and characterized by several spectroscopic and microscopic techniques. The characterization analysis revealed that the rGO-BC surface was decorated with the MnFe_2_O_4_. The strong chemical interaction (via electrostatic and H-bonding) between the integrated surface of rGO-BC and MnFe_2_O_4_ nanoparticles has been reported. The prepared composite was highly porous with a heterogeneous surface. The average size of the prepared composite was reported in the ranges of 2.6–7.0 nm. The specific surface area of the prepared composite was calculated to be 50.3 m^2^/g with a pore volume of 0.061 cc/g and a half pore width of 8.4 Å. As well, many functional sites on the nanohybrid composite surface were also found. This results in the excellent adsorption properties of nanohybrid composite and the effectual elimination of methylene blue dye from water. The nanohybrid was tested for various linear isotherms, such as Langmuir and Freundlich, for the adsorption of methylene blue dye. The Freundlich isotherm was the well-fitted model, proving the adsorption is multilayer. The maximum Langmuir adsorption capacity of nanohybrid composite for methylene blue was reported to be 74.627 mg/g at 27 °C. The adsorption kinetics followed the pseudo-second-order recommended surface interaction between the dye and nanohybrid composite. The interaction between methylene blue and the nanohybrid composite was also confirmed from the FTIR spectrum of the methylene blue-loaded adsorbent. The rate-determining step for the present study was intraparticle diffusion. Temperature-dependent studies of methylene blue adsorption were also carried out to estimate adsorption’s free energy, enthalpy, and entropy. The methylene blue adsorption was feasible, spontaneous, and endothermic. A comparison study revealed that the present materials could be successfully prepared and used for wastewater treatment.

## 1. Introduction

In the modern world, pollutants in water are one of the most significant environmental problems. As a result, the problem is only expected to worsen [1]. Most of these pollutants are, but not limited to, colored, containing dyes [2]. Dyes are generally complex heterocyclic organic structures that are chemically inert. These inert structures are responsible for the non-biodegradable properties and toxicity of the dye [3].

Among various dyes, methylene blue (MB) is one of the most commonly used dyes in textile industries [4]. MB is a cationic dye with a heterocyclic structure whose toxicity has been reported in the literature [5].

The human body has been reported to experience many harmful effects from MB dye, mainly stomach and chest pain, severe nausea and vomiting, skin irritation, high fever, pounding heartbeats, breathing difficulties, etc. [6,7,8]. As a result of MB consumption, the cardiovascular system, gastrointestinal tract, and central nervous system are also damaged [9].

To overcome these hazards, it is necessary to remove MB from the water before it is consumed by humans [9]. Among these efforts, adsorption technology has been found to be the most affordable, sustainable, and straightforward way of treating water [10]. Nanomaterials (NMs) have been considered to be better for this method. Conventionally, spinel-type ferrite NMs with a typical formulation, M-Fe_2_O_4_ (M = Mn, Fe, Co, Ni, and Cu), have widely been used for wastewater treatment [11,12]. Ferrites are magnetic NMs devices with numerous physiochemical and functional properties [13]. Besides having a high saturation magnetization, they have the advantage of being nanosized and having a high surface range-to-volume ratio.

Among these ferrites, manganese ferrite (MnFe_2_O_4_) is the most effective ferrite NMs due to its low cost, low toxicity, ease of preparation, high surface area, high mechanical hardness, excellent chemical stability, and bulky hydroxyl groups on their surfaces [14]. MnFe_2_O_4_ and its composites have been widely used in water treatment as adsorbents [15]. However, more efforts to increase the efficiency and sustainability of NMs are essential. The latest and most advanced attempt has been to modify the organic surface with ferrite NMs [12,16,17]. Therefore, incorporating NMs in the organic surface may also improve the thermal and mechanical properties of the organic material [18].

Of note, most graphene oxide (GO) or reduced GO (rGO) modifications were achieved with NMs [17,19,20]. GO has proven attractive for auxiliary metals and oxides of metals due to its high electrical conductivity, particular mechanical strength, and huge specific surface area [19]. However, such materials’ high cost and low efficiency still need to be solved. To overcome these problems, many other efforts have been made. The most useful among them is the merging of organic substances containing bulky functional groups, such as cellulose [21], chitosan [22], and biopolymer sponge [23], with GO. These organic/organic-integrated surfaces proved to be very useful for water treatment. However, this effort may be more helpful by making an integrated surface through a biochar/hydrochar or activated carbon (AC) of natural waste products [24,25].

Integrating these carbons with GO can be a good option, but producing these carbon materials requires high energy consumption and chemical activation [26,27]. This production further increases the cost of the overall process. Furthermore, gas evolved during the production of biochar/AC also becomes a problem for the air environment [28]. Therefore, moving towards natural plant material (without converting in AC or hydro/biochar) can be a successful attempt at sustainable, affordable material and will create more research opportunities for researchers [29].

Looking at the previous studies, it is found that many types of natural plant materials, like leaves [30,31,32] and seeds [33], etc., have been used (without converting in AC) to prepare nanocomposite followed by water treatment applications.

Microorganisms that float freely in water take nutritional signals from static surfaces, grow on them, and form biofilms that cause infectious diseases [34]. Gibert et al. [35] describe the biofilm development on the granule AC used for drinking water treatment. Bogino et al. [36] theorized that plant materials provide a suitable surface for bacterial growth. Therefore, a material with antimicrobial activities has been used in water treatment to overcome these shortcomings [34].

Additionally, these materials have high adsorption capacity and antibacterial activity. These materials have cellulosic surfaces with bulky functional groups that attract metal ions and lead to the formation of composites. Depending on adsorption, they can be the best water treatment option.

Among various natural materials, *Nigella sativa*, commonly known as black cumin (BC) seeds, displayed remarkable adsorption properties [37]. These seeds have been merged with several NMs, showing their usefulness for water treatment applications. BC seeds have an emerging antimicrobial activity that may help prepare antibiofilm nanocomposite for wastewater treatment [29]. The sucrose-functionalized magnetic BC seeds were utilized for the removal of Cr(VI) and Pb(II) ions and were found to be effective [38]. However, a rare study appears on merging two organic frameworks, such as GO–natural plant materials. In this study, the same effort is being made.

The study underpins an approach of growth of manganese ferrite (MnFe_2_O_4_) NPs onto the integrated surface of reduced graphene oxide (rGO) and the seed of plants of Black cumin (BC) (MnFe_2_O_4_/rGO-BC) via co-precipitation reaction. BC seeds’ (*Nigella Sativa*) surface is cellulosic with plenty of functional groups that can easily be modified [38]. GO is a highly oxidized form of graphene. It exists as an extended form of sheets. Functional groups are impregned to GO, such as -COOH, -C-O, C-O-C, and -OH groups. GO shows hydrophilic nature with high negative charge density. GO can also be modified easily [39,40]. Therefore, the fabrication of an integrated surface of GO-BC is easily possible. This integrated surface may respond to bulky functional groups on their surface, which may help in the growth of NPs on their integrated surface. The resultant hybrid nanocomposites may have a higher adsorption capacity of pollutants in the water that can be applied to remediate MB from water. This study aimed to prepare MnFe_2_O_4_/rGO-BC NMs and investigate their adsorption efficacy for MB dye in water. This research is the first reported material that has not been fabricated before, and the novelty of this study is to use an integrated organic surface prepared with natural food material.

## 2. Results and Discussion

### 2.1. Characterization Analysis

#### 2.1.1. FTIR Analysis

**Figure 1:**
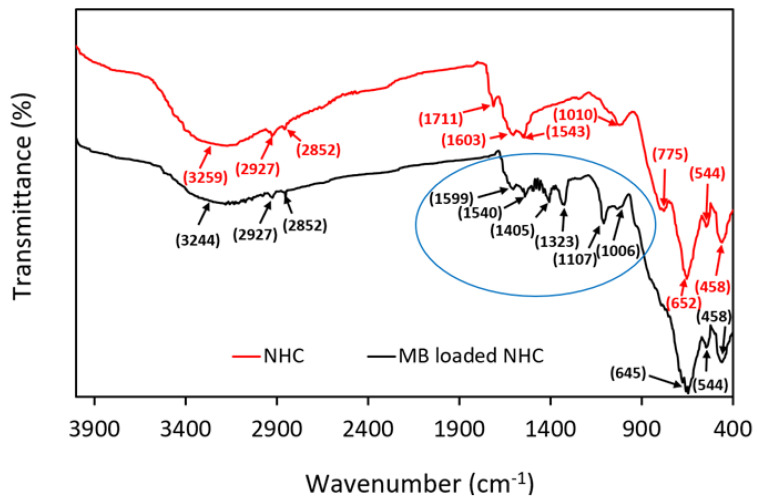
FTIR of MnFe_2_O_4_/rGO-BC NHC and MB-loaded NHC.

Figure 1 (red line) illustrates the FTIR analysis of the composite, and the figure shows the positions of the metal–oxygen bonds (Mn-O or Fe-O) [41].

The FTIR spectrum also shows the absorption bands for various functional groups present in the composite, with the major being -OH (hydroxyl) and C=O (carbonyl) groups. The reason for the presence of these groups in the composite is the integrated framework of rGO-BC seeds on which the NPs were grown [42]. -OH stretching vibration also refers to the moisture (water contents) present on the surface of the composite. It has been reported that the broad O–H stretching bands of water can overlap with the vibrational bands of the O–H stretching modes of alcohols and biomolecules [43,44].

Absorption peaks of amide I and II were also observed in the FTIR spectrum of the composite, confirming the protein structure of BC present in the composite [45]. All these peaks are detailed in Table 1.

To clarify the result, the FTIR spectrum of the prepared composite was compared with the FTIR spectra (Figure S1, Electronic Supplementary Information (ESI)) of virgin BC and rGO (Details given in Table 1) reported in our previous research [29]. These comparisons explain the growth of NPs on the integrated surface of rGO-BC through multiple shifting, as shown in Table 1.

The FTIR spectrum of MB-loaded adsorbent (NHC after MB adsorption) was also analyzed for this study. Figure 1 (black line) clearly shows the new absorption peak for MB, and the NHC peak is indicated in the blue circle. These peaks appear due to the heterocyclic structure of MB [46]. Additionally, a shift in the NHC functional groups’ peak and the vanishing of some peaks, such as the 1711 cm^−1^ peak, can also be observed in the FTIR spectrum of NHC after MB adsorption. All these peaks are listed in Table 1. According to this result, NHC showed a strong interaction with MB via electrostatic and non-electrostatic (H-bonding) interactions.

#### 2.1.2. X-ray Diffraction Analysis

**Figure 2:**
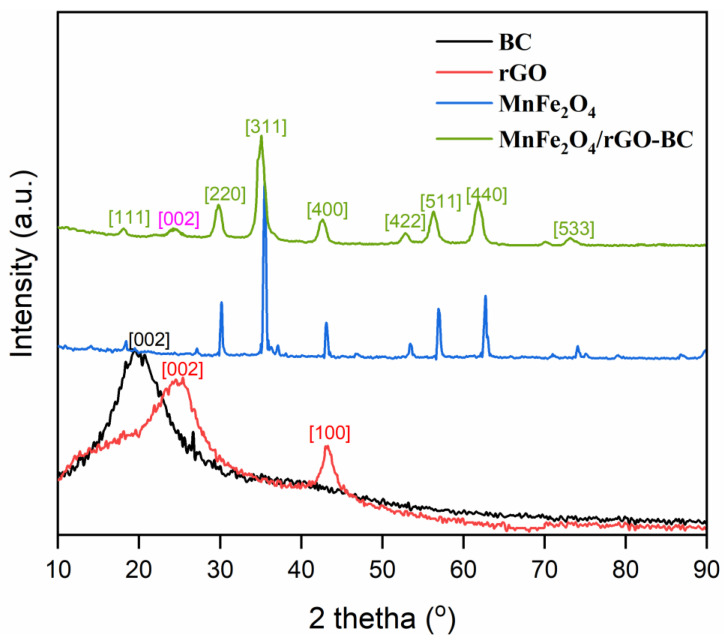
XRD of BC * (Reprinted with permission from Tara et al. [29], copyright (2021) Elsevier (License No. 5566540972617), rGO, MnFe_2_O_4_, MnFe_2_O_4_/rGO-BC NHC. * This study is an extension of our previous study [29], therefore, we obtained the copyright permission from the publisher.

Figure 2 characterizes the X-ray diffraction pattern of BC, rGO, MnFe_2_O_4_, and NHC. In Figure 2 (black line), the broad peak corresponding to the [002] plane suggests the cellulosic carbon surface of BC seeds [29]. The existence of rGO is shown by the presence of 2θ peaks at 24.55° and 43.16°, corresponding to the [002] and [100] planes in Figure 2 (red line), respectively [29]. The diffraction pattern of the bare MnFe_2_O_4_ (Figure 2, blue line) exhibited various peaks at 18.29°, 30.06°, 35.40°, 37.04°, 43.06°, 53.48°, 56.85°, 62.74°, and 74.09° (2θ) corresponding to the [111], [220], [311], [222], [400], [422], [511], [440], and [533] planes, respectively [41,47,48]. These positions of the peaks were also found in the diffraction pattern of NHC (Figure 2, green line) which matched the standard data of manganese ferrite XRD pattern (JCPDS card, file No. 73–1964). In the XRD pattern of the NHC (green line), the peak of MnFe_2_O_4_ corresponding to the [222] plane at 37.04° (in blue line) disappeared and the intensities of other peaks reduced. In addition, the peaks of rGO and BC moderated significantly as the NPs grew on the surface of rGO-BC [49]. The existence of a carbon framework (due to rGO-BC) in the NHC is shown in Figure 2 (green line) by the presence of a 2θ peak at 24.33° corresponding to the [002, in pink color] plane [29]. The current analysis shows similarity with previous study confirms that the material fabricated in this study is MnFe_2_O_4_ loaded rGO-BC [41,47,48]. 

#### 2.1.3. Microscopic Analysis of NHC

**Figure 3:**
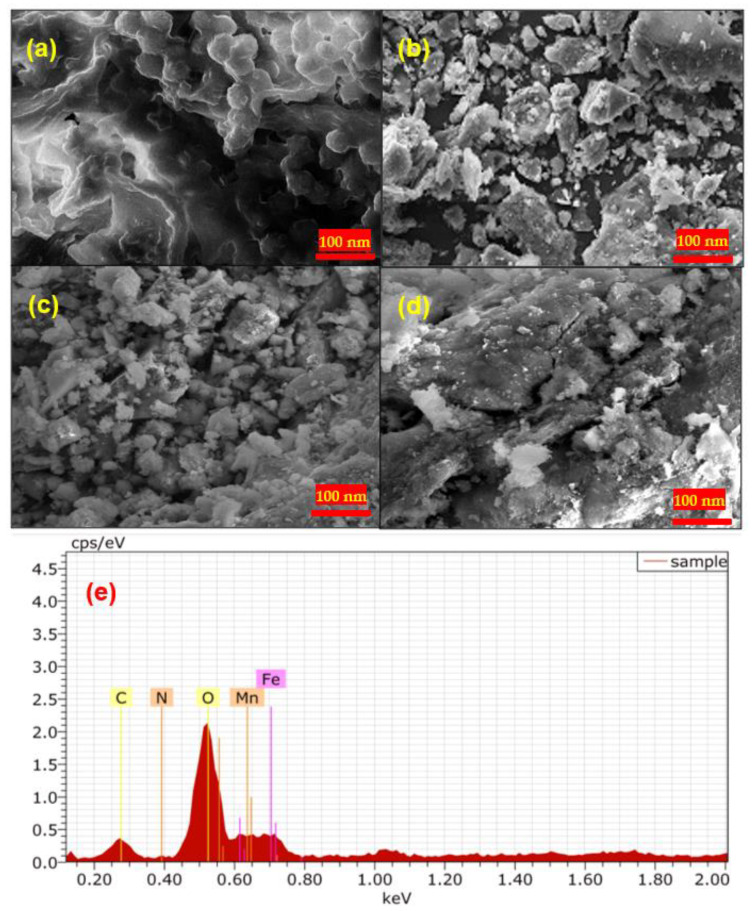
Results of SEM images of (**a**) pure black cumin seed powder, (**b**) rGO, (**c**,**d**) MnFe_2_O_4_/rGO-BC NHC, and (**e**) EDX analysis of MnFe_2_O_4_/rGO-BC NHC.

Herein, the SEM images of pure BC seed (Figure 3a), rGO (Figure 3b), and NHC (Figure 3c–e) have been discussed. The SEM images for NHC (Figure 3c,d) represented at different magnifications revealed that MnFe_2_O_4_ NPs have grown on the integrated surface of rGO-BC. The irregular shapes of NHC particles of various sizes can be seen in the FE-SEM image (Figure 3c,d), suggesting a heterogeneous structure of NHC. From the SEM images of NHC, it can be demonstrated that NPs are firmly intact on the integrated surface of rGO-BC [50].

SEM-EDX of the prepared composite (Figure 3e) was also analyzed, suggesting the presence of elements of C, O, N, Fe, and Mn. The EDX analysis indicates the elemental composition of C (13.57 weight %), O (41.20 weight %), N (2.97 weight %), Fe (21.35 weight %), and Mn (20.91 weight %) present in the NHC. The C, N, and O came from the plant seed and rGO, and Fe and Mn came from the ferrite NPs present in the NHC. The EDX analysis further confirmed the nanocomposite preparation by combining rGO-BC and MnFe_2_O_4_.

**Figure 4:**
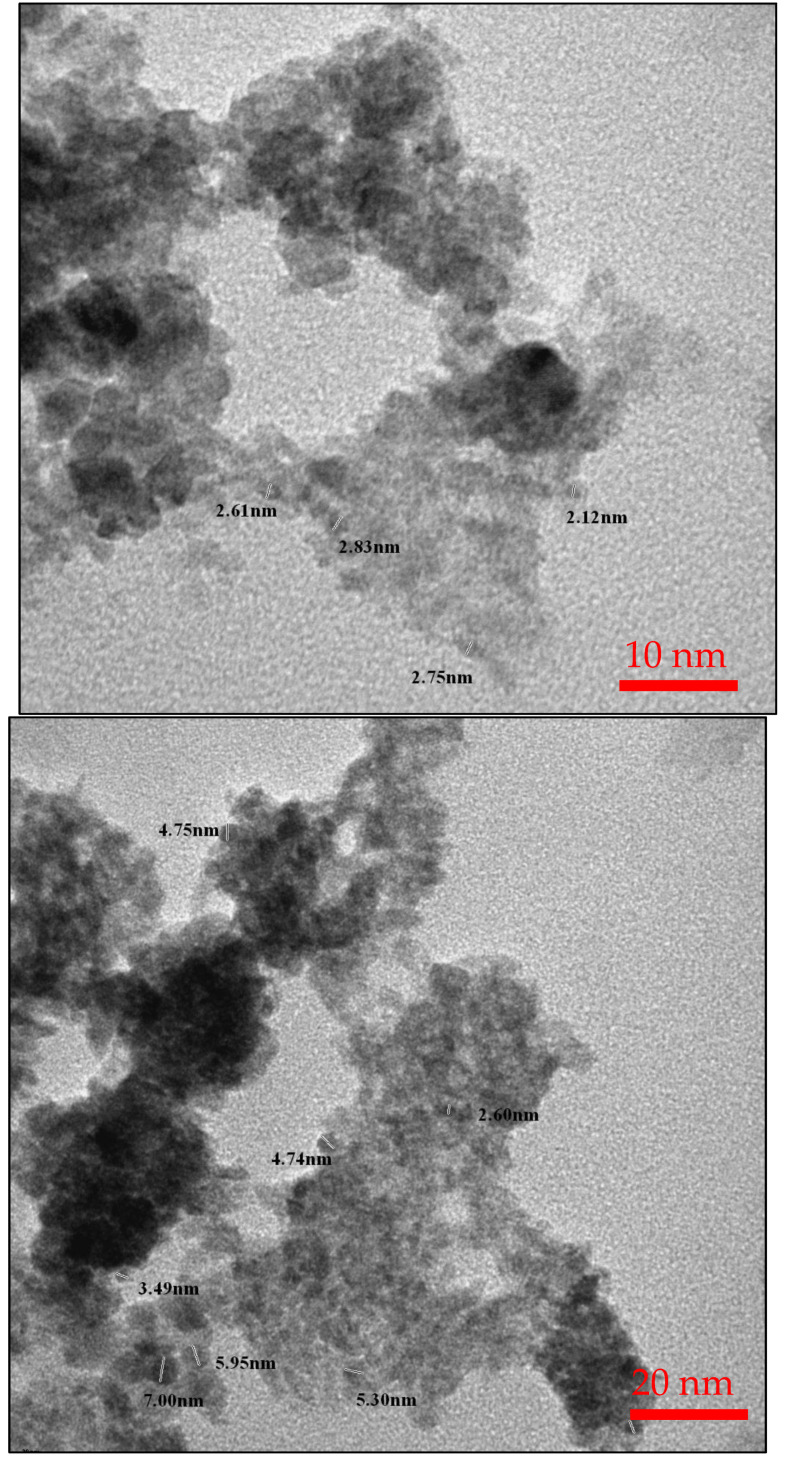
TEM images of MnFe_2_O_4_/rGO-BC NHC at different magnifications.

The morphology of the NHC was also studied using TEM, as shown in Figure 4. Figure 4 shows the morphology of NHC at different magnifications, confirming that rGO-BC was covered with the NPs. It is noted that NPs had good dispersion on rGO, showing a thin layer structure and folding character, which was inherited from rGO [51,52]. The TEM images demonstrated the NHC with an average size of incorporated NPs ranging between 2.6–7.0 nm.

#### 2.1.4. Thermogravimetry Analysis of NHC

**Figure 5:**
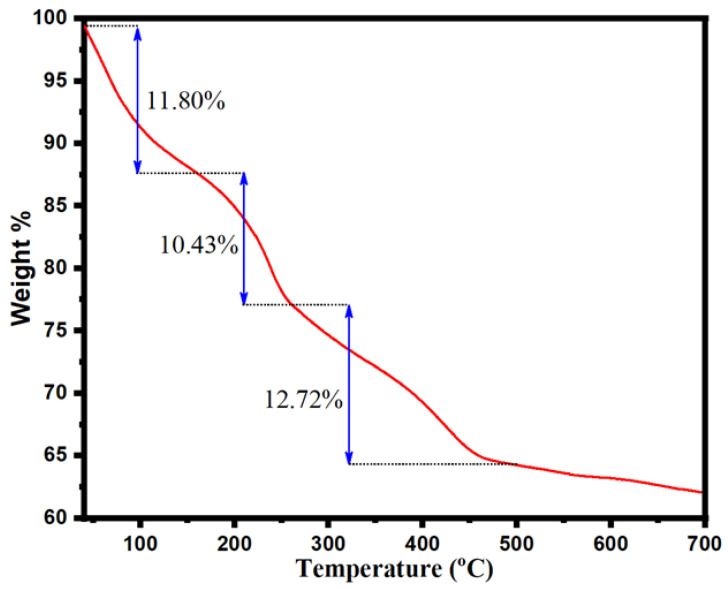
Result of thermal gravimetric analysis (TGA) of MnFe_2_O_4_/rGO-BC NHC.

The thermogravimetry analysis, TGA curve in the ranges from 30–800 °C, under the nitrogen atmosphere, is shown in Figure 5. The TGA curve reveals the three weight loss phases (moisture evaporation, main volatilization, and continuous volatilization) via pyrolysis of the carbon framework of NHC at a specific temperature range. A significant first-stage weight loss was observed (11.80 wt.%) at low temperatures (43 °C to 160 °C), ascribed to the moisture evaporation from the NHC. The second characteristic step of weight loss has shown in the interval temperature ranges from 160 °C to 260 °C (10.43 wt.%), while the third weight loss stage was observed from 260 to 500 °C (12.72 wt.%). These are accredited to the main volatilization and continuous volatilization via the decay of oxygen-containing groups (carbonyl, carboxyl, and hydroxyl) as well as the carbon framework of NHC [53,54]. The N_2_-containing solid residue may also have been produced under the N_2_ atmosphere [53]. These findings suggest 34.95 wt.% losses during pyrolysis. Therefore, the TGA curve represents good thermal stability of present NHC.

#### 2.1.5. Nitrogen Physisorption Study

**Figure 6:**
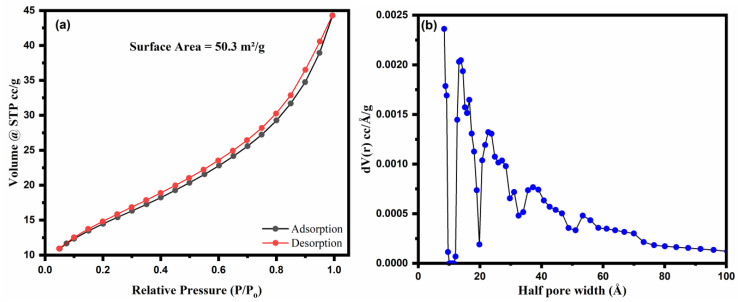
(**a**) N_2_ adsorption–desorption isotherms and (**b**) pore size distribution patterns of MnFe_2_O_4_/rGO-BC NHC.

The textual properties of the NHC used in this study can be examined from the given figures of N_2_ physisorption (Figure 6a,b). The type III isotherm was used for this study, describing the adsorbate’s interaction with the adsorbent surface. Type III isotherm plots can be explained via the H3 hysteresis loop according to the IUPAC classification. The H3 hysteresis loop can be seen in the present plot (Figure 6a), representing N_2_ adsorption by capillary condensation. This condensation may be due to the dispersion of heterogeneous aggregates containing the pores. For its clarity, the pore size distribution (PSD) pattern was extracted through the NLDFT model. The results of the PSD pattern (Figure 6b) clearly describe the macropores in the existing sample NHC. The nitrogen physisorption analysis revealed the specific surface area of the NHC that was calculated to be 50.3 m^2^/g with a pore volume of 0.061 cc/g and a half pore width of 8.4 Å, respectively.

#### 2.1.6. Zero-Point Charge Analysis

**Figure 7:**
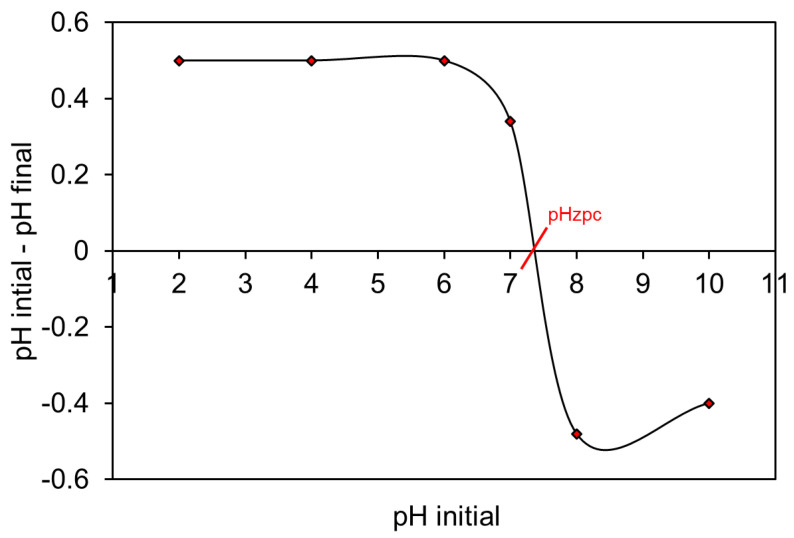
Result of zero-point charge analysis of MnFe_2_O_4_/rGO-BC NHC.

Zero-point charge (pHzpc) analysis was performed by the well-known salt addition method [55]. The details of the method adopted are given in the Electronic Supplementary Information (ESI). The pHzpc result can be seen in Figure 7, which shows that the pHzpc for the NHC adsorbent is approximately 7.5. It can be concluded from pHzpc that an aqueous solution pH below 7.5 gives a protonated positive NHC surface, whereas above 7.5, it gives a negatively charged surface in solution. This means that the alkaline pH of the solution (above 7.5) will provide a better environment for NHC to adsorb the cationic MB dye.

### 2.2. Results of Adsorption Studies

Batch adsorption experiments were supported to understand the behavior of adsorption of MB dye onto the NHC surface under the impact of variable experiment parameters. In the batch experiments, 10 mL of MB solution, having an initial concentration of 10 mg/L, was used as a solute solution.

#### 2.2.1. Adsorbent Amount and pH Effect

The adsorbent amount optimization experiments were carried out with 0.5, 1.0, 1.5, 2.0, 2.5, and 3.0 g/L of NHC for 10 mL of 10 mg/L of MB solutions, which were stirred for 120 min at 27 °C (300 K). The effect of the adsorbent amount on the adsorption percentage can be seen in Figure S2a (Electronic Supplementary Information (ESI)). The minimum amount of adsorbent (0.05 g/L) could remove 93% of MB from its given concentration. The adsorption % was increased to ~97.0% with the increase in NHC to 1.0 g/L. It is evident that with the increase in the adsorbent dosage, the entire surface sites available for adsorption will be increased; thus, the adsorption of pollutants on the increased adsorption sites will also be increased for fixed solute concentration [56]. The higher adsorption % of both pollutants with a minimum amount of NHC suggests the high efficiency of NHC towards MB dye.

The pH of the solution affects the degree of surface charge and speciation of the adsorbate molecules and, thus, ultimately affects the removal efficiency of the adsorbent [57]. For this study, the effect of pH on the adsorption efficiency of NHC was observed in pH range 2–10. A significant increase in the MB adsorption onto the NHC surface can be seen in the direction of alkaline pH (range 8–10) from acidic pH (range 2–6). The maximum adsorption of MB dye was obtained at pH 10 (Figure S2b, Electronic Supplementary Information (ESI)).

The MB (cationic) dye molecules are positively charged and can easily be attracted toward the negative surface. The pHzpc of present NHC was measured to be approximately 7.5 (Figure 7). It has also been reported that present NHC has bulky functional groups such as -OH, -C=O, and -COOH. Therefore, these functional groups are deprotonated at alkaline pH (pH value higher than pHzpc) during adsorption and acquire a negative charge [58]. This deprotonation increases the electrostatic interaction between the deprotonated functional groups of NHC and cationic MB dye molecules. Thus, the alkaline solution pH favored MB dye adsorption onto the NHC surface [59]. In this study, the maximum adsorption of MB was found at a higher pH range of 8–10.

#### 2.2.2. Temperature Effect, Thermodynamics, and Isotherms

**Figure 8:**
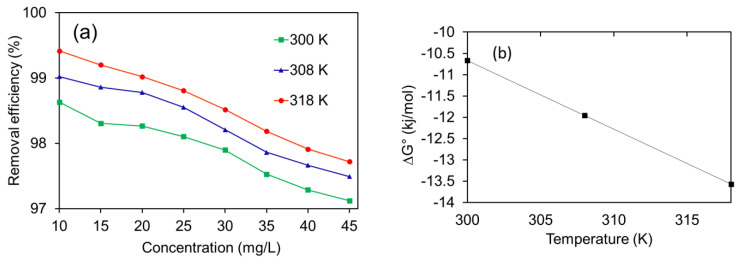

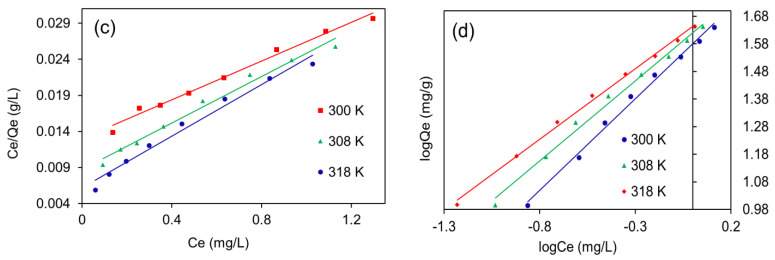
Results of (**a**) temperature effect, (**b**) thermodynamic plot, (**c**) Langmuir isotherm, and (**d**) Freundlich isotherm for MB adsorption on the MnFe_2_O_4_/rGO-BC NHC.

The temperature effects on the removal efficiency of NHC for MB dye were investigated in range 27 to 45 °C (300–318 K) temperatures range at the fixed concentrations of MB in solution (10 mg/L) and 1.0 g/L of NHC adsorbent dosage (Figure 8a). The better adsorption performance for this study was observed at a higher temperature, i.e., 45 °C. That means the reaction was endothermic as the adsorption performance of NHC increased with temperature [60]. At higher temperatures, the dye diffusion rate increases; therefore, the present trend was obtained [60].

By applying the obtained temperature-dependent MB adsorption data, the thermodynamic equation (Equations (3) and (4)) gave the parameters such as Gibbs free energy change (∆G°), enthalpy change (∆H°), and entropy change (∆S°) (Table S1, Electronic Supplementary Information (ESI)) [61].

(1) ∆G°=RT lnK

(2) ∆G°=∆H°−T∆S°

where Kc (equilibrium constant) = Qe/Ce.

These parameters were utilized to study the effect of temperature on the adsorption capacity of the NHC.

The −*Ve* values of ΔG° = −10.67 to −13.57 kJ/mol at 27–45 °C temperatures (Figure 8b), respectively, confirmed the feasibility and spontaneity of the present adsorption process in the given temperature range [62]. The interactions of MB to functional groups of NHC {ΔG° (adsorption) for MB = ΔG°_NHC_ + ΔG°_NHC---MB_ + ΔG°_BC----MB_ + ΔG°_rGO---MB_ + ΔG°_MnFe2O4---MB_} attributed to the overall ΔG° values.

The increase in negative values of ∆G° on increasing the temperature represented the high feasibility of adsorption upon raising the temperature (Figure 8a). This can be accounted for based on the increased diffusion rate of MB onto the NHC surface at elevated temperatures. This also suggested that the process would be endothermic.

The endothermic nature of MB adsorption can also be confirmed by the +*Ve* value of ΔH° = +37.69 kJ/mol. The enthalpies of solute–solid interactions decrease as the solute molecules are solvated in a liquid–solid system. The decrease means that energy is needed to displace the solvent (H_2_O) from the solid surface and desolvate the MB molecules from the solution (MB——H_2_O), which leads to the positive change in enthalpy (endothermic process) of the reaction [63].

The +*Ve* value of ΔS° = +0.016 kJ/mol/K confirmed randomness at the solid–liquid interface for the MB system [63]. The desolvated MB and the displaced solvent (H_2_O) both gains extra translational energy, leading to a rise in entropy and increasing the liquid–solid system’s unpredictability [63]. Moreover, the interaction of MB with the functional groups of NHC (-C-O-——MB/-OH——-MB) and protonation and deprotonation of the functional groups (-OH and -C=O) present on the NHC surface are also to blame for the positive value of ∆S°. FTIR analysis for NHC post-adsorption can support such interactions.

For obtained temperature-dependent MB adsorption data, isotherms models were also verified. Isotherm parameters are the most important ones to explain the adsorption mechanism at the solid–liquid interface. Isotherms can also be used to determine the maximum adsorption capacity. The common isotherm models Langmuir and Freundlich were used in this study to explain MB’s adsorption mechanism onto the NHC surface. The homogenous adsorbent surface where a monolayer of adsorbate forms is suitable to the Langmuir isotherm [64]. The Langmuir isotherm equation in Table 2 can be used to calculate the maximal monolayer adsorption capacity, Q_o_. The Freundlich isotherm is applicable for an adsorbent surface that is heterogeneous and on which a multilayer of adsorbate is produced. The equation in Table 2 can be used to get the Freundlich parameters, *k_F_* values, and *n* values.

The result of Langmuir isotherm can be reported as follows. The R^2^ values of Langmuir plots were found to be lower than Freundlich isotherm and deviate from unity at a temperature range 27 to 45 °C, respectively, suggesting poor fitting of MB adsorption data to Langmuir isotherm (Figure 8c). Although the Langmuir model did not fit the MB adsorption data, their parameters give important information about the present MB adsorption process. The maximum MB adsorption capacity of NHC from Langmuir isotherm was calculated to be 74.626 mg/g at 27 °C. The higher adsorption performance might be due to the bulky functional groups on the NHC surface. The separation constant R_L_ values were obtained close to zero (0.088–0.033), recommending the feasibility of the MB adsorption process at all temperature ranges.

The Freundlich result is reported as follows to verify the MB adsorption data further. The R^2^ values of Freundlich plots were found to be higher than the Langmuir isotherm and close to unity at all the temperature ranges, suggesting well-fitting MB adsorption data to the Freundlich isotherm (Figure 8d). The Freundlich isotherm gave the *n* (heterogeneity measurement) values in the range 1–10 at 1.509, 1.725, and 1.957, suggesting the heterogeneous behavior of MB adsorption. The heterogeneous surface of NHC can be confirmed from the SEM image of NHC (Figure 3c), which is responsible for the present heterogeneous MB adsorption process. The k_F_ values (Freundlich constant) at 38.00, 41.696, and 43.984 (mg/g) (L/mg)^1/n^, at a temperature range of 27 to 45 °C, respectively. Again, the overall increase in the *k_F_* value suggests the endothermic nature of MB adsorption onto the NHC surface. Therefore, the MB adsorption was multilayered on the heterogeneous surface of NHC and endothermic. Similar result was also reported for previous study [65].

#### 2.2.3. Optimization of Contact Time, Adsorption Kinetics, and Mechanism

**Figure 9:**
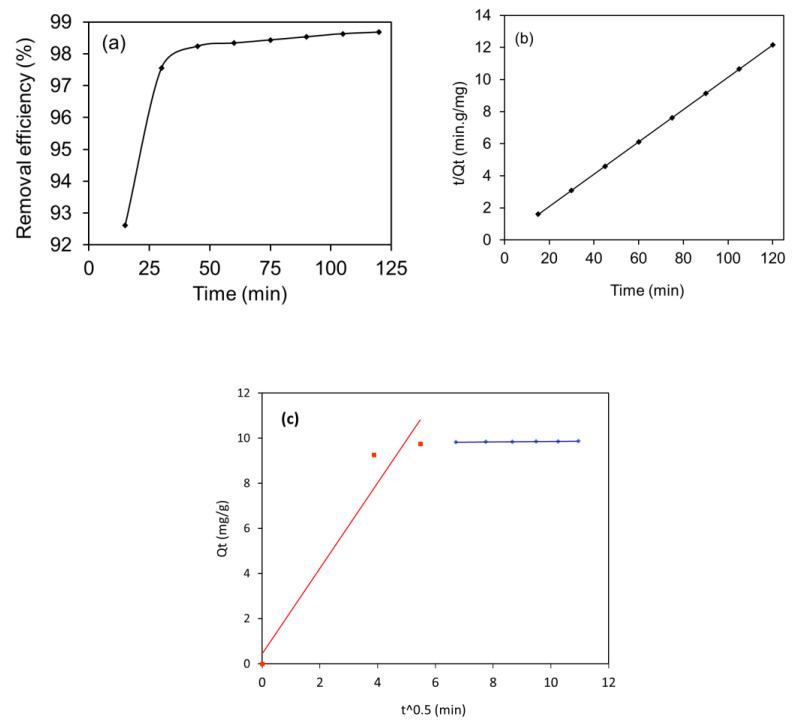
Results of (**a**) contact time effect, (**b**) pseudo-second-order kinetic, and (**c**) Weber–Morris plot for MB adsorption on the MnFe_2_O_4_/rGO-BC NHC.

The contact time optimization was achieved by agitating the 10 mL of MB solution sample (having a concentration of 10 mg/L of MB and 1.0 g/L of NHC amount) for 120 min at a time interval of 15 min (Figure 9a). The experimental results indicate that 92% of MB was eliminated within 15 min and equilibrated at 45 min, where approximately 99% of MB was removed. As suggested by the FTIR spectrum, there were massive adsorption sites on the surface of NHC (Figure 1). These sites were initially free for MB adsorption (within 15 min) and thus showed a fast removal rate. After the specific time (15 min), there may be only specific free sites on the NHC surface [66]. Therefore, the reaction might slow down after 15 min and finally be saturated. This result also suggests that the present process of MB adsorption was completed in several steps. These steps may control and determine the adsorption rate. Generally, the adsorption rate depends on the physiochemical properties of the adsorbent.

To better understand the adsorption rate, two kinetic models, pseudo-first-order and pseudo-second-order, were applied to the above time-dependent adsorption data. These two well-known kinetic models gave important kinetic parameters for MB adsorption [67].

The pseud-first-order model assumes that the adsorption rate depends on the unoccupied adsorptive sites of the solid surface. The physical bond formation between adsorbent and adsorbate is the rate-limiting step for pseudo-first-order.

The pseudo-second-order model assumes that the adsorption rate depends on the solid surface’s unoccupied adsorptive sites and adsorbate molecules in the liquid phase. The chemical bond formation between adsorbent and adsorbate is the rate-limiting step for pseudo-second-order.

These models are supported by the mathematical equations given in Table 3. To test the compatibility of the models with the experimental adsorption data acquired, the coefficients of determination (R^2^) and parameters of the kinetic models were determined according to Table 3. The pseudo-first-order model (Figure S2c, Electronic Supplementary Information, ESI) gave a large difference between the theoretical (1.346 mg/g) and experimental value (9.804 mg/g) of *Qt* (adsorption capacity at the given time) (Table 3). In addition, pseudo-first-order gave the R^2^ value at 0.91 for its linear plot and the rate constant, *K*1, value of 0.05089/min. These experimental results suggest the unsuitability of the pseudo-first-order model to the time-dependent MB adsorption data.

For the pseudo-second-order model (Figure 9b), the R^2^ value was found to be close to unity (0.99) along with the *k*_2_ value = 0.138 (g/mg/min). In addition, the theoretical value of *Qt* obtained from the pseudo-second-order shows good agreement with the value of experimental *Qt* (Table 3). These results indicate that the pseudo-second-order model is well suited for the present MB adsorption study. Better fitting of the pseudo-second-order model suggests operation of the present adsorption process via chemisorption [68].

The time-dependent MB adsorption data were further verified by using Weber–Morris kinetic model to demonstrate the rate-determining (slow) step [69]. The existing Weber–Morris plot (Figure 9c) that does not pass through the origin confirms that the present MB adsorption process was multi-step. Furthermore, two straight lines indicate a two-step adsorption process (red line: 0–30 min and blue line: 45–120 min). Actually, in this model, there is a confluence of two plots, film diffusion (first line) and intraparticle diffusion (second line), and the determination of parameters of these plots (*Kd*_1_, *C*_1_ for film diffusion and *Kd*_2_, *C*_2_ for intraparticle diffusion) gives information about the actual rate-determining step. The intercept value of the line is directly proportional to the boundary thickness, while it is inversely proportional to the adsorption rate. Therefore, the smaller slope (kd) and larger line intercept reflect the rate-determining step. As for the present study, the two lines {first (red) line for film diffusion and second (blue) line for intraparticle diffusion} can be seen in the Weber–Morris plot, in which the value of intercept (*C*_2_) was found to be more significant for the second line (Table 3), which reflects that the MB adsorption rate in the current adsorption process was governed by particle diffusion. A similar observation was reported by Elkady et al. [69].

In summary, the present MB adsorption process follows pseudo-second-order kinetics combined with intraparticle diffusion, suggesting that the MB adsorption process operates via chemisorption and is limited by intraparticle diffusion. The possible mechanisms suggested by the FTIR spectrum of the MB-loaded NHC (Figure 1b) were electrostatic interactions and non-electrostatic interaction, as summarized in Section 2.1.1 [65].

#### 2.2.4. Regeneration and Reutilization

**Figure 10:**
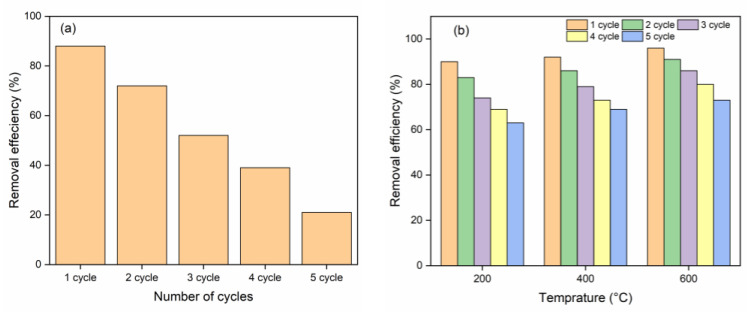
Results of reutilization of MnFe_2_O_4_/rGO-BC for MB adsorption via (**a**) chemical treatment and (**b**) thermal treatment.

Figure 10a shows that, after chemical regeneration and reusing the NHC for several cycles, the NHC exhibits very good removal efficiency for the first two cycles and then abruptly drops in the third cycle. By the fifth cycle, removal efficiency was reduced to approximately 20%. Hence, the current absorbent holds up better to two cycles after chemical treatment.

Figure 10b reflects thermal regeneration and reusability of exhausted NHC for MB adsorption. The results show that NHC maintains a very good removal efficiency for each cycle, with a slight decrease in the removal efficiency at each temperature. It can be further seen from Figure 10b that the removal efficiency of the regenerated NHC increases with the increase of the heating temperature. With the fifth reusable cycle, the removal efficiencies of NHC for MB dye were recorded as 63, 69, and 73% at 200, 400, and 600 °C, respectively. The decomposition of MB dye on the surface of NHC may be the reason for the increase of removal efficiency with heating temperature. The adsorption sites (or pores) are filled upon adsorption of MB, and after heating, the adsorbed MB decomposed to carbon content. The decomposition of the MB increases with increasing temperature, and at higher temperatures the carbon content evaporates, and then fresh adsorption sites (or pores) are responsible to restore the adsorption efficiency of thermally treated NHC [70,71,72]. This result suggests the NHC to be a better adsorbent from an economic point of view.

#### 2.2.5. Comparative Analysis

Various functional groups were found on the surface of developed NHC, and the experimental procedures and regeneration results proved that the present NHC is a better adsorbent for MB dye. However, for any adsorbent to be of practical importance, it is necessary to compare its adsorption capacity with other existing adsorbents. In particular, the adsorption capacity depends on various experimental conditions for the adsorbent such as temperature, pH, adsorbent dosage, contact time, and dye concentration. In these experimental conditions, the comparative study of adsorbents does not prove to be very meaningful, but the bias remains. In these experimental conditions, to reduce this biasness, a parameter called partition coefficient (PC) was introduced, which proved to be quite meaningful [73].

In earlier studies, the PC was studied and it was reported that PC gave the less biased and real capacity performance of any adsorbent. Therefore, for the present study also, the PC was derived to estimate the actual performance of NHC under different conditions (Table 4). The PC value was derived by using Equation as given below [73].

(3) PC=Adsorption capacity/Final concentration.

(4) PC=Adsorption capacity/initial concentration × removal rate.

It can be estimated from Table 4 that the value of PC for the current study (i.e., 72.0 L/g) is much higher than those of several other adsorbents. The maximum adsorption capacity and equilibrium adsorption capacity value of the present NHC adsorbent is also higher than other adsorbents, although the dosage of the present adsorbent was significantly lower than that of others, which reflects the essence of the present adsorbent. Compared to the acid-washed black cumin seeds, the present NHC adsorbent provided almost similar maximum adsorption capacity and equilibrium adsorption capacity with the same adsorbent dosage, however, the PC value for the present NHC adsorbent was higher than that of the acid-washed black cumin seeds. The contact time was also reported to be longer for acid-washed black cumin seeds than for the NHC adsorbent. So, it can be said that the present adsorbent performed better than other adsorbents which may prove to be a better option for wastewater treatment in the future.

## 3. Experimental

### 3.1. Materials and Methods

The chemicals used in the present study are highly pure and of analytical grade. The black cumin seeds (*Nigella sativa*) were purchased from a local market in New Delhi, India. All chemicals, including Manganous sulfate monohydrate (MnSO_4_·H_2_O, 99%), Ferric chloride (Anhydrous) (FeCl_3_, 96%), Graphite flakes (99.9%), Hydrochloric acid (HCl, 37%), Sodium hydroxide (NaOH, 97%), Hydrogen peroxide (H_2_O_2_, 30%), and Methylene blue (C_16_H_18_ClN_3_S, 97%) were purchased from Sigma Aldrich are highly pure and of analytical grade.

### 3.2. Synthesis of Reduced Graphene Oxide (rGO)

The rGO from graphite powder was prepared by Hummers’ process, similar to that reported in our previous study [29]. In a typical batch synthesis, the desired amount of graphite flakes (2.0 g) and sodium nitrate (1.0 g) in 50 mL of concentrated H_2_SO_4_ (98%) was energetically stirred for half an hour in an ice bath to avoid the suspension temperature being lower than 0 °C. After that, 6.0 g of KMnO_4_ was added slowly to the above suspension. Then, the ice bath was removed, and the mixture was stirred at 10 °C for 24 h. The reaction mixture was then cooled and converted into a dark green colored paste. To this, 300 mL of cooled deionized water was added, and the suspension temperature was quickly increased to 90 °C, which was maintained by an external heating system for 5–10 min. Bubbles were then obtained in the reaction mixture. The reaction mixture was stirred for an additional 3 h. The reaction was terminated by adding cooled deionized water, 60 mL of 30% H_2_O_2_ solution, and 200 mL of 10% HCl solution, and thus, suspension color changed to yellow. Afterward, the solid particles were centrifuged with 10% HCl solution and deionized water. GO particles were found after drying under a vacuum at 60 °C for 24 h. This obtained GO sample was then exfoliated to rGO by drying the sample at 250 °C for 2 h. The prepared sample was characterized as rGO.

### 3.3. Preparation of NHC

Black Cumin (BC) seeds were cleaned with double distilled water and dried overnight at 60 °C in the oven. Then, fine powder was obtained by using the grinding machine. The rGO was synthesized by the above-reported method. The preparation of MnFe_2_O_4_/rGO-BC was carried out via a simple co-precipitation process. Briefly, 0.5 g of prepared rGO was dispersed into 100 mL of deionized water using ultrasonication. Another beaker had also been used for the dispersion of black cumin seed powder (0.5 g) in 100 mL of deionized water under ultrasonication. After that, both solutions were mixed and stirred for 1 h on a magnetic stirrer. After that, 1.622 g of FeCl_3_·6H_2_O and 0.98 g of MnSO_4_·H_2_O were dissolved in deionized water (100 mL), separately, and then mixed with the above solution. The reaction mixture was continuously agitated at 80 °C for half an hour. After that, a solution of 8 M NaOH was added to increase the pH of the mixture to 10.5. The mixture was sustained for 15 min and cooled down at room temperature. The black product was isolated by centrifugation for 5 min at 5000 rpm and washed with acetone and deionized water to eliminate unreacted content. The particles of the nanohybrid composite (NHC) were then dried at 60 °C for 48 h to obtain a fine powder. We have also described a similar synthesis scheme to our earlier study in which MnO_2_ was incorporated on the integrated surface of rGO and BC seeds [29]. Some other previous literature also reported similar preparation patterns for various NMs [17,30,31,32,33].

### 3.4. Preparation of Adsorbate Stock Solution

The MB dye stock solution was made by dissolving 1000 mg of MB dye in 1000 mL of distilled water. Using the rule of dilution, the produced solution was diluted to meet the specifications.

### 3.5. Characterization and Instrumentations

Various spectroscopic and microscopic techniques were used to characterize the prepared sample. The instruments used for characterization analysis are given in the Electronic Supplementary Information (ESI).

### 3.6. Adsorption Experiments

The adsorption experiments in a batch piece of equipment were carried out. Different process parameters such as contact times (15–120 min), adsorbent amount (5–30 mg/L), initial solution pHs (2.0–10.0), initial concentrations (10–45 mg/L), and temperatures (30–50 °C) were optimized in this study. During these experiments, the concentrations of pollutants in solution before and after adsorption experiments were determined spectrophotometrically using a UV-Vis spectrophotometer.

For this spectrophotometric measurement, 3.5 mL of the MB sample was taken in the Type1 UV10 (square) quartz cuvette of 10 mm path length containing 4.5 mL solution. These concentrations were used to determine the adsorption performance of NHC in the form of adsorption capacity and removal efficiency as (Equations (1) and (2)), respectively:

(5) $$\mathrm{Adsorption}\;\mathrm{capacity}\;Q_{e}=(C_{o}-C_{e})\frac{V}{m},$$

(6) $$\mathrm{Removal}\;\mathrm{efficiency}\;(\%)=\left(\frac{C_{o}-C_{e}}{C_{o}}\right)100.$$

Here, the *Q_e_* (mg/g): the equilibrium adsorption capacity, the *C_o_* (mg/L): the initial concentration, the *C_e_* (mg/L): the equilibrium concentration, the *V* (L): the volume of dye solution, and the *m* (g): mass of adsorbent.

The MB adsorption data were also verified by the adsorption kinetics, thermodynamics, and isotherm models at given experimental conditions. The formulations included in these models are detailed in their respective tables.

### 3.7. Regeneration and Reutilization

Using adsorbent in multiple cycles dramatically reduces the cost of the adsorption process and increases the adsorbent’s overall efficiency. The adsorbent was regenerated and reused in several cycles to test its efficiency. Two processes, chemical and thermal, were used for adsorption regeneration in this study under the optimum obtained conditions. For regeneration procedures (chemical and thermal), 2 g of NHC was mixed with 1000 mL of MB dye solution at 10 mg/L up to saturation. The MB-loaded NHC was then separated from the dye solution and dried in a hot air oven at 90 °C for 24 h.

For the chemical treatment, the 1 g of MB-loaded NHC was added to 100 mL of 0.5 mol HCl and stirred for 8 h, after which it was separated and washed several times with deionized water so that the pH became neutral, and then it was dried at 90 °C for 24 h.

For thermal treatment, 1 g MB-loaded adsorbent was heated at three temperatures, 200, 400, and 600 °C, in muffle furnace (under the limited oxygen environment) for 2 h. The thermally treated sample was cooled overnight at room temperature [70,71,72]. After that, the treated NHC was thoroughly washed with deionized water to eliminate the carbonized material left over the NHC, and then dried in a hot air oven at 90 °C for further use.

Under optimal conditions, the regenerated (thermally and chemically) NHCs were used for the next adsorption cycle. This adsorption–desorption cyclic process was carried out for five cycles. The efficiency of the reutilized adsorbent was measured spectrophotometrically.

## 4. Conclusions

In this study, the manganese ferrite particles incorporated the functional groups containing the surface of reduced graphene oxide–black cumin seeds. This newly reported nanohybrid composite was synthesized by a simple co-precipitation method and characterized through various analyses such as FTIR, X-Ray Diffraction, SEM-EDX, TEM, N_2_ adsorption, zero-point charge, and thermal analysis. The synthesized composite was investigated for its adsorption performance in a batch manner. The nanohybrid composite has shown multilayer adsorption for methylene blue dye, which was confirmed by the fitting of the Freundlich isotherm. The higher Langmuir adsorption capacity was calculated as 74.627 mg/g at 27 °C. Pseudo-second-order kinetics was suggested for this study, which suggested the interaction between the active sites of the composite surface and dye molecules. The interaction between the nanohybrid composite and methylene blue was also confirmed from the FTIR spectrum of the methylene blue-loaded nanohybrid composite. This study emphasizes that natural plant material, which is inexpensive, can be used as an alternative to activated carbon. Due to the cellulose carbon surface of natural plant materials, they can be easily modified, and affordable composites can be developed. These composite materials can be beneficial for adsorption applications due to having cellulose surfaces. Therefore, the present nanohybrid composite may be strategically advantageous for removing pollutants from water. A study of this adsorbent’s antibiofilm activity should be conducted soon, as well as an investigation of its use in real water systems. It is also necessary to determine the efficiency of this adsorbent for adsorbing other pollutants to develop better technology.

## Figures and Tables

**Table 1:** List of FTIR bands and related functional groups assigned for MnFe_2_O_4_/rGO-BC NHC and MB-loaded NHC at various wavenumbers.

Order	Wavenumber Assigned for BC * (cm^−1^)	Corr. Peaks	Wavenumber Assigned for rGO * (cm^−1^)	Corr. Peaks	Wavenumber Assigned for MnFe_2_O_4_/ rGO-BC (cm^−1^)	Corr. Peaks	Wavenumber Assigned for MB-Loaded MnFe_2_O_4_/ rGO-BC (cm^−1^)	Corr. Peaks
1.	3368	-OH str.	3316	-OH str.	3400–3250	-OH str.	3244	-OH str.
2.	2922, 2853	-C-H str.	2917, 2855	-C-H str.	2927, 2852	-C-H str.	2927, 2852	-C-H str.
3.	1706	-C=O str.	1589	C=C str.	1711	-C=O str.	-	-
4.	1657	Amide I	-	-	1603	Amide I	1599	Amide I
5.	1549	Amide II	-	-	1543	Amide II	1540	Amide II
6.	1410	-OH bend	-	-		-	1405–1100	Heterocyclic structure of MB
7.	1259	C-O str.	1388	-OH bend	1010	-OH bend	1006	-OH bend
8.	1100–700	-C-O-C str. and/or C-C str.	1090	C-O str. and/or C-O-C str.	544, 458	M-O str. and/or M-O-M str.	544, 458	M-O str. and/or M-O-M str.

* This study is an extension of our previous study [29], therefore, we obtained the copyright permission from the publisher.

**Table 2:** The isotherms parameters for the adsorption of MB dye onto MnFe_2_O_4_/rGO-BC.

Temp (°C)	Langmuir $\frac{C_{e}}{Q_{e}}=\frac{C_{e}}{Q_{o}}+\frac{1}{Q_{o}b}$				Freundlich $\log Q_{e}=\log k_{F}+\frac{1}{n}\log C_{e}$		
	Monolayer Adsorption Capacity, Q_o_ (mg/g)	Langmuir Constant (Adsorption Intensity), b (L/mg)	Separation Constant, R_L_	Regression Coefficient, R^2^	Freundlich Constant (Multilayer Adsorption Capacity), k_F_ (mg/g) (L/mg)^1/n^	Freundlich Constant (Heterogeneity), n	Regression Coefficient, R^2^
27	74.627	1.030	0.088	0.987	38.000	1.509	0.993
35	62.111	1.850	0.051	0.984	41.696	1.725	0.992
45	55.866	2.890	0.033	0.980	43.984	1.957	0.997

**Table 3:** Kinetics parameter for adsorption of MB dye onto rGO-BC@MnFe_2_O_4_.

Kinetics	Mathematical Equation	Parameters	Results Experimental Adsorption Capacity, *Q_t_*, exp = 9.804 mg/g
Pseudo-first-order	$\log(Q_{e}-Q_{t})=\log Q_{e}-\frac{k_{1}}{2.303}t$	Pseudo-first-order constant, *k*_1_ (1/min)	0.051
		Pseudo-first-order adsorption capacity at time *t*, *Q_t_*, cal (mg/g)	1.348
		Regression coefficient, R^2^	0.916
Pseudo-second-order	$\frac{t}{Q_{t}}=\frac{1}{h}+\frac{t}{Q_{e}}$	Pseudo-second-order constant, *k*_2_	0.138
		Pseudo-second-order adsorption capacity at time *t*, *Q_t_*, cal mg/g	9.940
		Regression coefficient, R^2^	0.999
Weber–Morris model		First (red) line, *Kd*_1_	1.893
	$Q_{t}=k_{ipd}t^{0.5}+C$	First (red) line, *C*_1_	0.436
		Second (blue) line, *K*_2_	0.107
		Second (blue) line, *C*_2_	9.175

**Table 4:** Comparative results of MB adsorption studies with various adsorbents *. Adapted with permission from Abdulla et al. [34], copyright (2020) Elsevier (License No. 5575340013674).

Order	Adsorbent	Adsorbent Dose (g/L)	Contact Time (min)	Solution pH	Temperature (°C)	Initial Concentration (mg/L)	Maximum (Langmuir) Adsorption Capacity (mg/g)	Equilibrium Adsorption Capacity (mg/g)	Removal Efficiency (%)	PC (L/g)	Ref.
1.	Ag-Ag_2_O/ZrO_2_/GL	2.0	30.0	7.0	27.0	10.0	43.9	4.9	99.0	50.6	[34]
2.	Acid-washed black cumin seeds	1.0	60.0	7.0	27.0	10.0	73.5	9.8	98.1	51.6	[59]
3.	MnFe_2_O_4_/BC	3.0	45.0	7.0	27.0	10.0	10.1	3.3	99.4	52.6	[74]
4.	CuO	2.5	30.0	6.0	22.0	25.0	64.0	5.5	54.7	0.5	[75]
5.	Haloxylon recurvum plant stems	4.0	40.0	8.0	25.0	20.0	22.9	4.8	96.4	6.7	[76]
6.	Solanum tuberosum plant leaves	2.0	24.0	7.0	30.0	10.0	52.6	3.9	79.0	1.9	[77]
7.	Fe_2_O_3_-SnO_2_/BC	2.0	90.0	7.0	27.0	10.0	58.8	4.9	97.9	23.9	[78]
8.	MnFe_2_O4/rGO-BC	1.0	45.0	7.0	27.0	10.0	74.6	9.9	98.6	72.0	This study

* We have extracted the data from previous study [34], therefore, we obtained the copyright permission from the publisher.

## Data Availability

All the data and materials related to the manuscript are published with the paper and available from the corresponding author upon request.

## Supplementary Materials

The following supporting information can be downloaded at: https://www.mdpi.com/article/10.3390/molecules28135007/s1. Characterization and instrumentation analysis; Zero-point charge determination of NHC; Figure S1: FTIR spectra of reduced graphene oxide, rGO and black cumin seeds, BC; Figure S2: Results of (a) adsorbent dose effect, (b) pH effect, (c) pseudo first order kinetic for MB adsorption on to the Mn-Fe_2_O_4_/rGO-BC NHC; Table S1: Results of thermodynamic parameters at various temperatures

## Sample Availability

Samples of the compounds are available from the authors.
