# Supplementary material for: Nigella sativa-Manganese Ferrite-Reduced Graphene Oxide-Based Nanomaterial: A Novel Adsorbent for Water Treatment
Source: Molecules. 2023 Jun 26;28(13):5007. doi: 10.3390/molecules28135007 (PMC10343191; doi:10.3390/molecules28135007)
Supplement: Supplementary file 1 [file molecules-28-05007-s001.zip › molecules-2389710-supplementary.pdf]

Electronics Supplementary Information, ESI

Characterization and instrumentation analysis

Analysis	Instrument	Company detail	Country	Remarks
pH of solution	pH meter	Model number APX 175 E/C, Control dynamics instrumentation Pvt. Ltd.,	India	pH of solution was adjusted by using acidic (0.1M HCl) and/or basic (0.1M NaOH) solution
Crystal phase and size	X-ray diffractometer	Ultima IV, Rigaku	United State	Condition: 2θ range 10-80º, Cu-Kα radiation (λ =1.54 Å), a Cu-filter, generator voltage 35 kV, 30mA current, and a proportional counter detector
Functional groups	Spectrometer	Shimadzu	Japan	Analyzed in mid IR range, i.e., 4,000-400 cm^{-1}
Surface morphology	Scanning electron microscope	Nova nano SEM 450, FEI	United State	-
Elemental composition	Energy-dispersive X-ray spectroscope	Bruker 127 eV	United State	-
Particle shape and size	Transmittance electron microscope	F30 S-Twin, Tecnai microscope	United State	Operated at 80 kV.
Thermal analysis		Perkin Elmer Pyris 6	United Kingdom	
Absorbance	UV—Vis spectrophotometer	T80-UV/VIS, PG instruments Ltd	United Kingdom	-
Surface area and pore size	Surface area and pore size analyzer (Measurement by Gas Sorption)	Autosorb iQ-MP/XR (Quantachrome)	United State	-
Zeta potential	Zeta Potential Analyzer	Malvern Panalytical Ltd	United Kingdom	At 25 °C with count rate 138.8

Zero-point charge determination of NHC

The zero-point charge of the NHC was measured by the salt addition method as shown by the flow chart below.

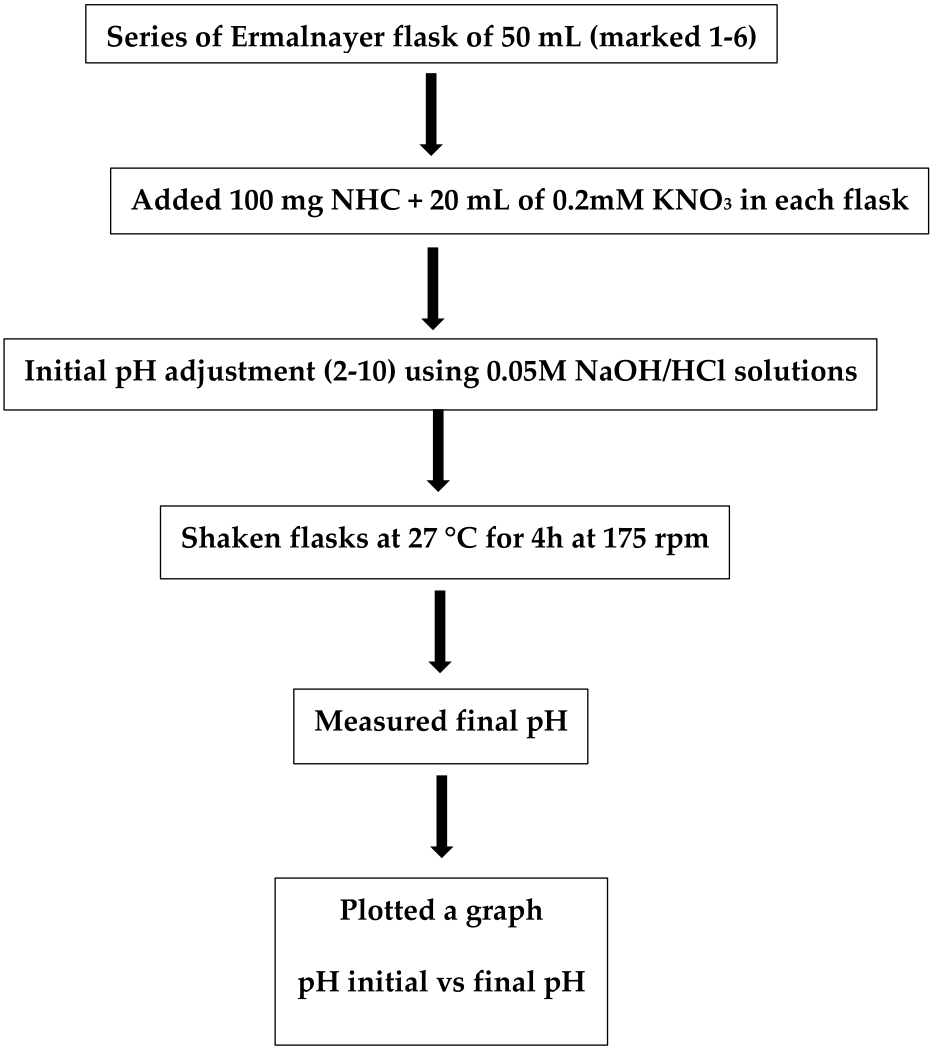

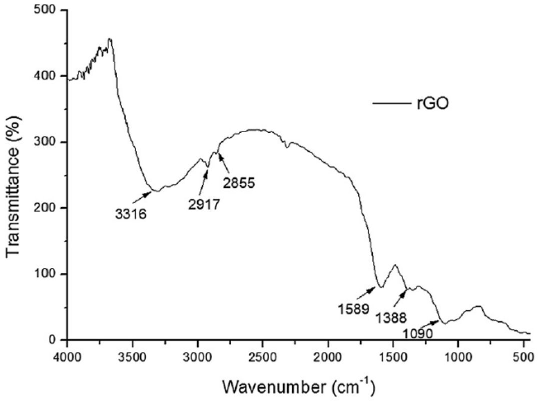

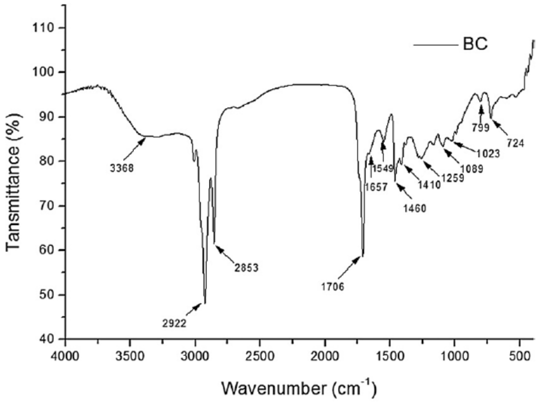

Figure S1. FTIR spectra of reduced graphene oxide, rGO (Reprinted with permission from Tara et al [29], copyright (2021) Elsevier (License No. 5566540972617) and black cumin seeds, BC (Reprinted with permission from Tara et al [29], copyright (2021) Elsevier (License No. 5566540972617).

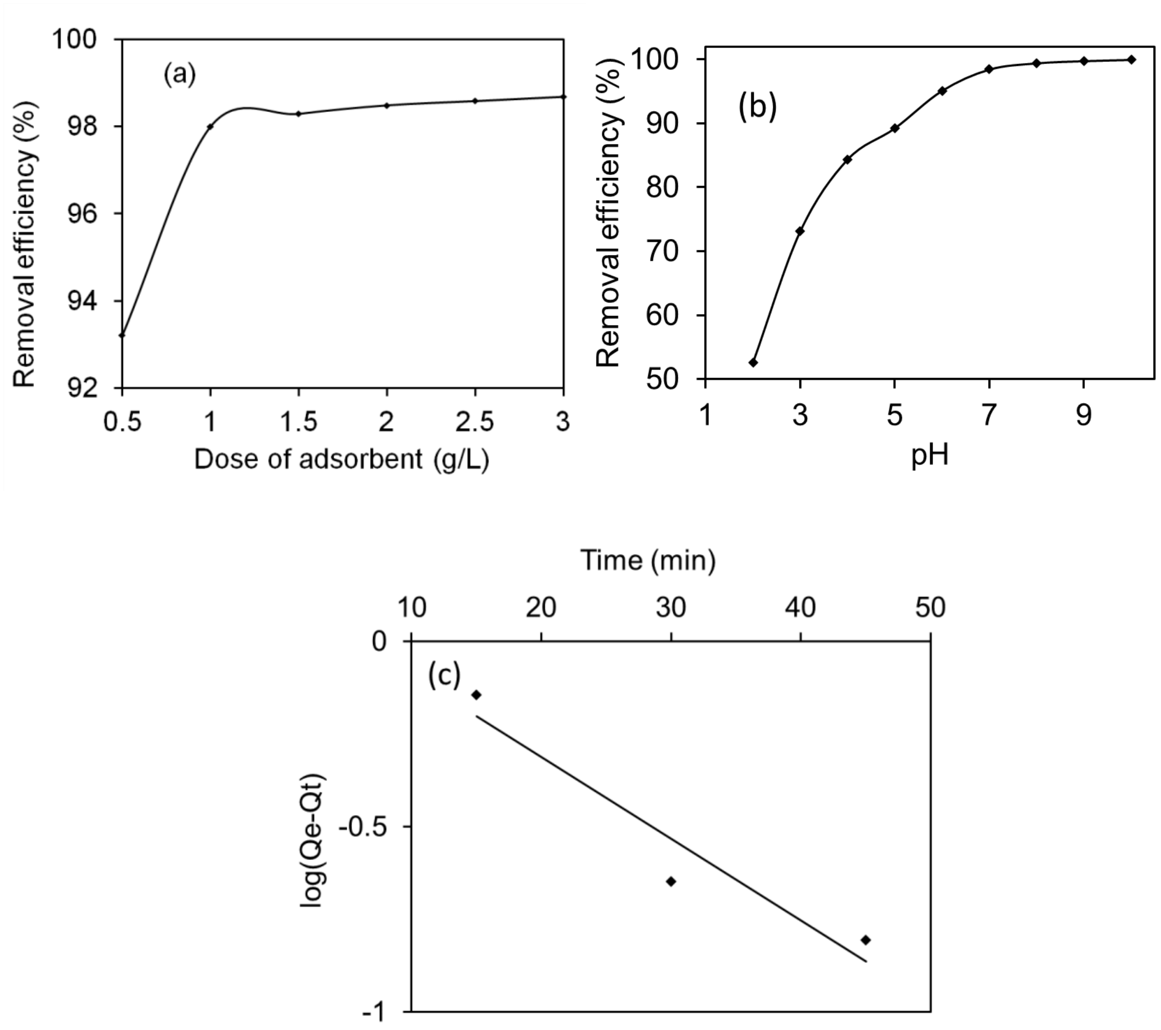

Figure S2. Results of (a) adsorbent dose effect, (b) pH effect, (c) pseudo first order kinetic for MB adsorption on to the Mn-Fe_2O_4/ rGO-BC NHC.

Table S1. Results of thermodynamic parameters at various temperatures

Temperature (K)	ΔG (KJ/mol)	ΔH (KJ/mol)	ΔS (KJ/mol/K)
300	-10.673	+37.687	+0.161
308	-11.962		
318	-13.574		
